# Supplementary material for: Cis-regulatory atlas of primary human CD4+ T cells
Source: BMC Genomics. 2023 May 11;24:253. doi: 10.1186/s12864-023-09288-3 (PMC10173520; doi:10.1186/s12864-023-09288-3)
Supplement: Supplementary file 5 — Additional file 5. [file 12864_2023_9288_MOESM5_ESM.pdf]

Supplemental Figures and Figure Legends

Supplemental File 1: Supplemental Figures and Figure Legends

- Figure S1: Expression of Nearest Genes to Cis-Regulatory Elements
- Figure S2: ChIP-Seq of Previously Proposed Silencer Enriched Histone Marks.
- Figure S3: ChIP-Seq Performed in CD4+ T Cells.
- Figure S4: Cis-Regulatory Enrichment Within Super-Enhancers
- Figure S5: Cis-Regulatory Element Histone ChIP-Seq Enrichment with Nucleosome-Positive and Nucleosome-Negative Clustering.
- Figure S6: Silencer Database (SilencerDB) Intersection of Cis-Regulatory Elements.
- Figure S7: CRE Location and CpG Promoter Enrichment.

Supplemental Table 1: Primers and dsDNA sequences used for luciferase validation: *All sequences of primers and dsDNA used in this study.*

Supplemental Table 2: CRE locations described by Lenti-STARR-Seq: *Locations of genome-wide significant CRE in MACS2 narrowPeak format.*

1. Final significant NREs
2. Final significant Enhancers

Supplemental Table 3: Utilized datasets: *Identifiers of all external utilized datasets, including ENCODE and other publications.*

Supplemental Table 4: Read Count and Statistics Summary: *Read count statistics and unique read counts of each hCD4+ T cell Donor replicate.*

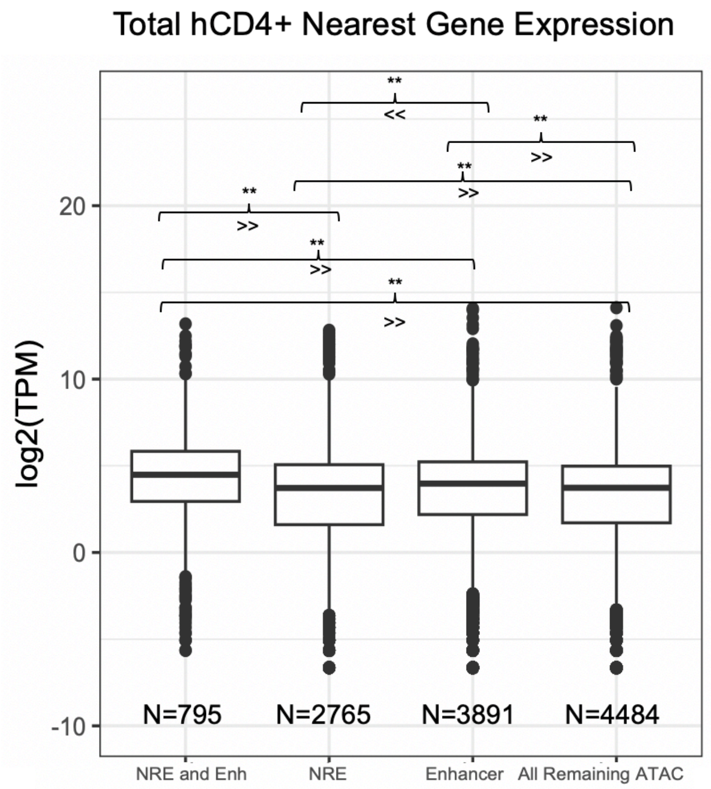

Supplemental Figure 1: Expression of Nearest Genes to Cis-Regulatory Elements. Transcripts per million (TPM) in human (h)CD4+ PolyA RNA-Seq at TSS within 10 kb of genes with either both Enhancer and NREs, Enhancer only, NREs only, or 'All' ATAC-Seq peaks from input. Boxplot displays median and interquartile range. [** Adjusted P-value ≤ 0.05 by non-parametric Kruskal-Wallis one-sided test with Holm adjustment for multiple comparisons].(1)

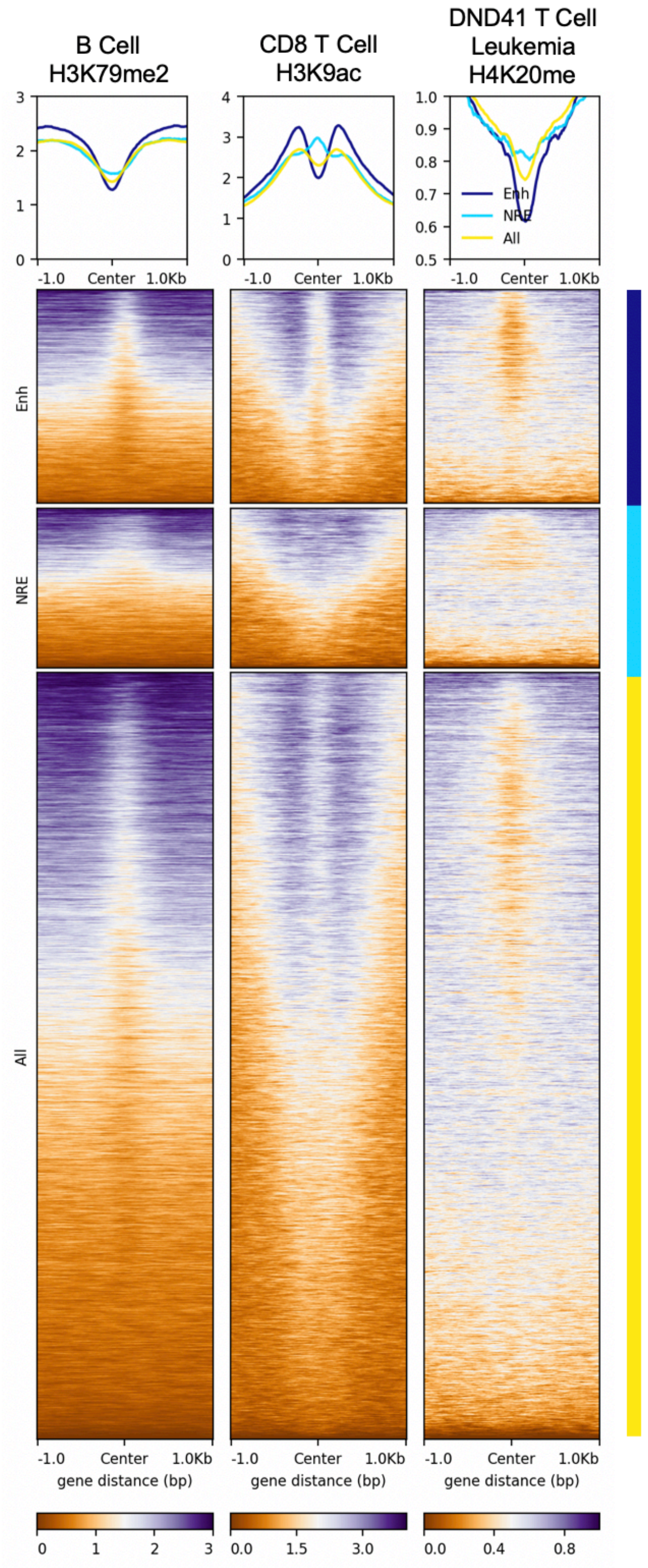

Supplemental Figure 2: ChIP-Seq of Previously Proposed Silencer Enriched Histone Marks. Enrichment of transcription factors or histone ChIP-Seq experiments in non-CD4+ T cells. The selected histones demonstrate enrichment at NREs (silencers) in other silencer screens. (1–3)

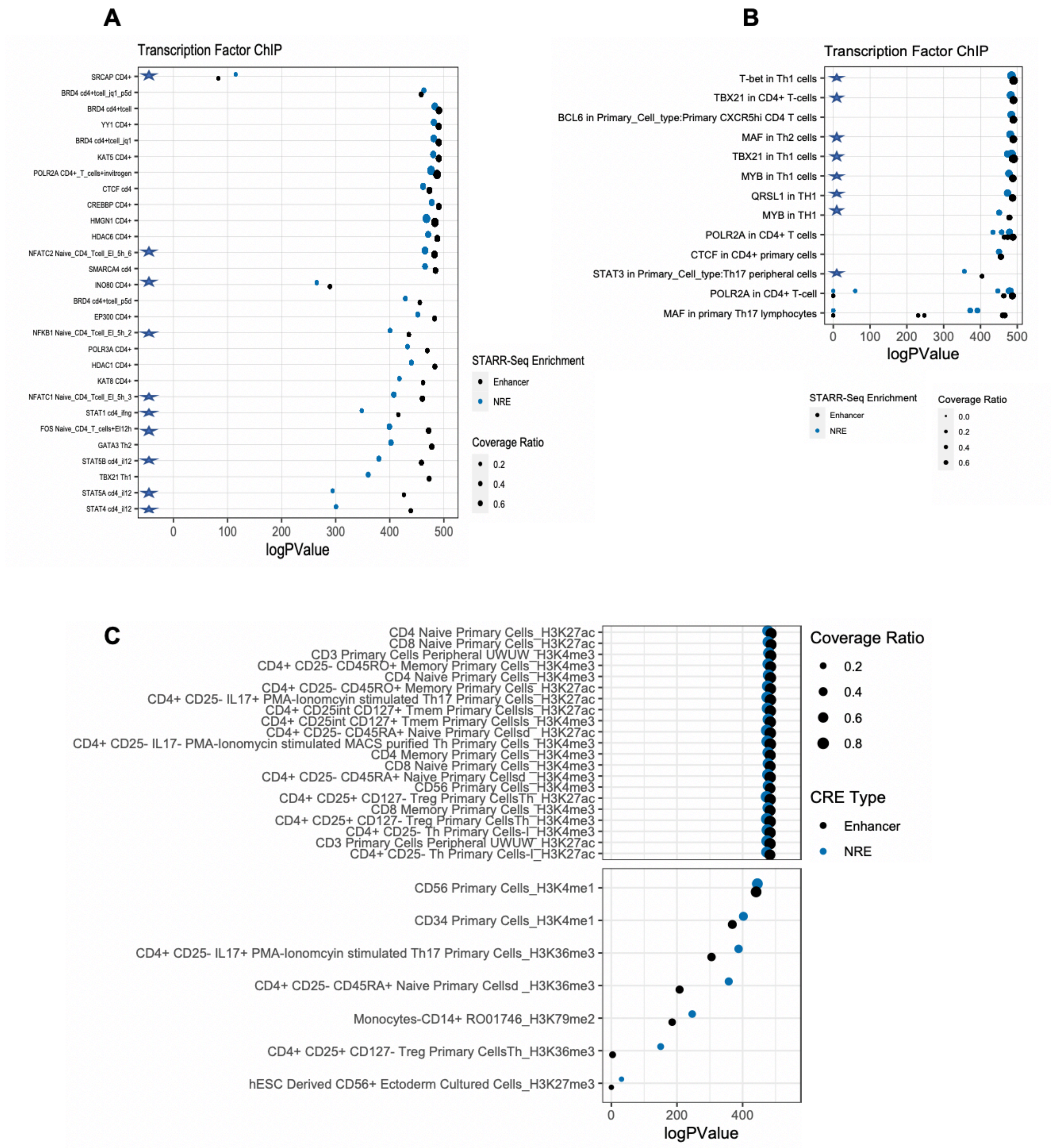

Supplemental Figure 3: ChIP-Seq Performed in CD4+ T Cells. RELI enrichment LogP-value of ChIP-Seq experiments performed in human CD4+ T cells across STARR-Seq–identified Cis-regulatory elements (CREs). Star indicates the experiment was performed in activated cells. **A)** Datasets in RELI and **B)** Datasets from GEO, and **C)** Histone modification ChIP-Seq enrichment in GEO.(4)

A

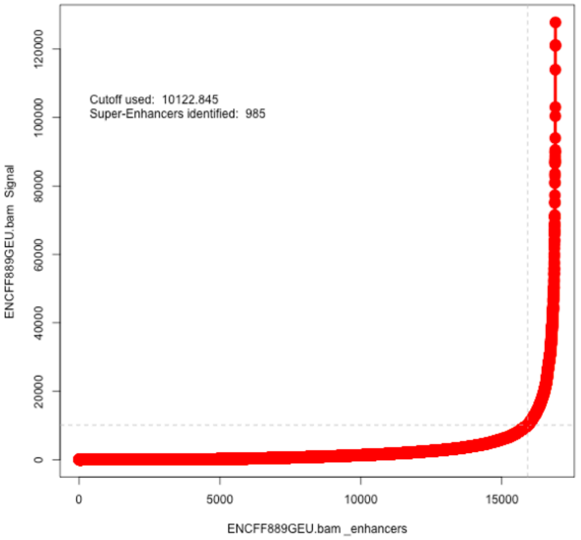

B

Chi-Squared p-value	ATAC-Seq All Peaks
Enh	1.481e-13
NRE	0.1811

Supplemental Figure 4: Cis-Regulatory Enrichment Within Super-Enhancers A) Super-enhancer ranks are called using ROSE algorithm with H3K27ac ChIP-Seq experiment performed in hCD4+ T cells.(1,5) **B)** P-value of 2x2 Chi-squared test for the difference in the number of overlaps of super-enhancers with enhancers [or negative regulatory elements (NREs)] versus all ATAC-Seq input peaks.(1)

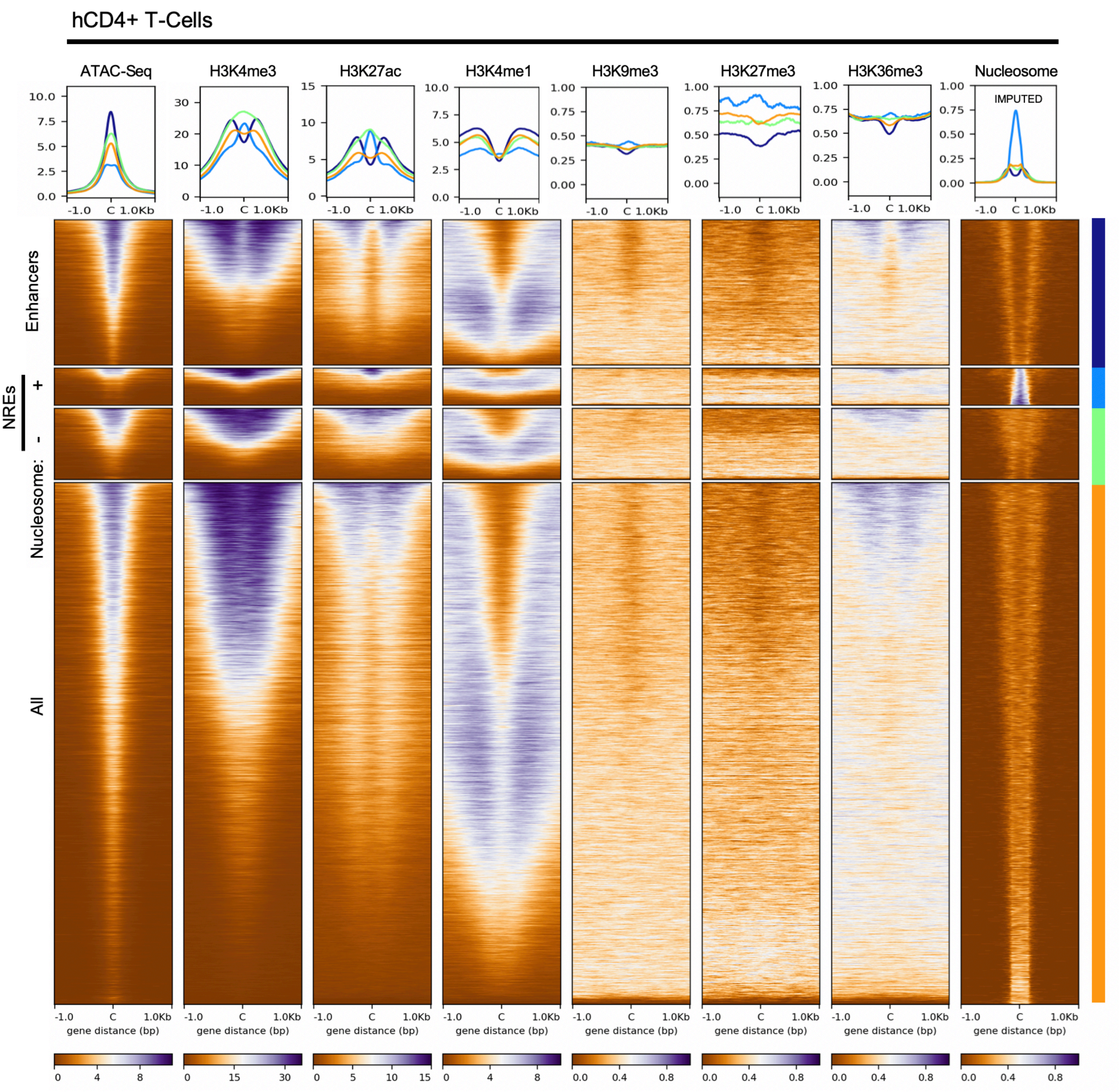

Supplemental Figure 5: Cis-Regulatory Element Histone ChIP-Seq Enrichment with Nucleosome-Positive and Nucleosome-Negative Clustering. Tag density plots of mean intensity and heatmaps of ChIP-Seq or ATAC-Seq performed in human CD4+ T cells plotted against STARR-Seq–identified Enhancers, negative regulatory elements (NREs), and 'All' open peaks from input.(1) Nucleosome location in resting CD4+ T cells imputed from NucleoATAC.(6) Tag density of mean signal intensity displayed for histone enrichment and of imputed nucleosome position. NRE sites only are k-means (n=2) clustered by imputed nucleosome signal.

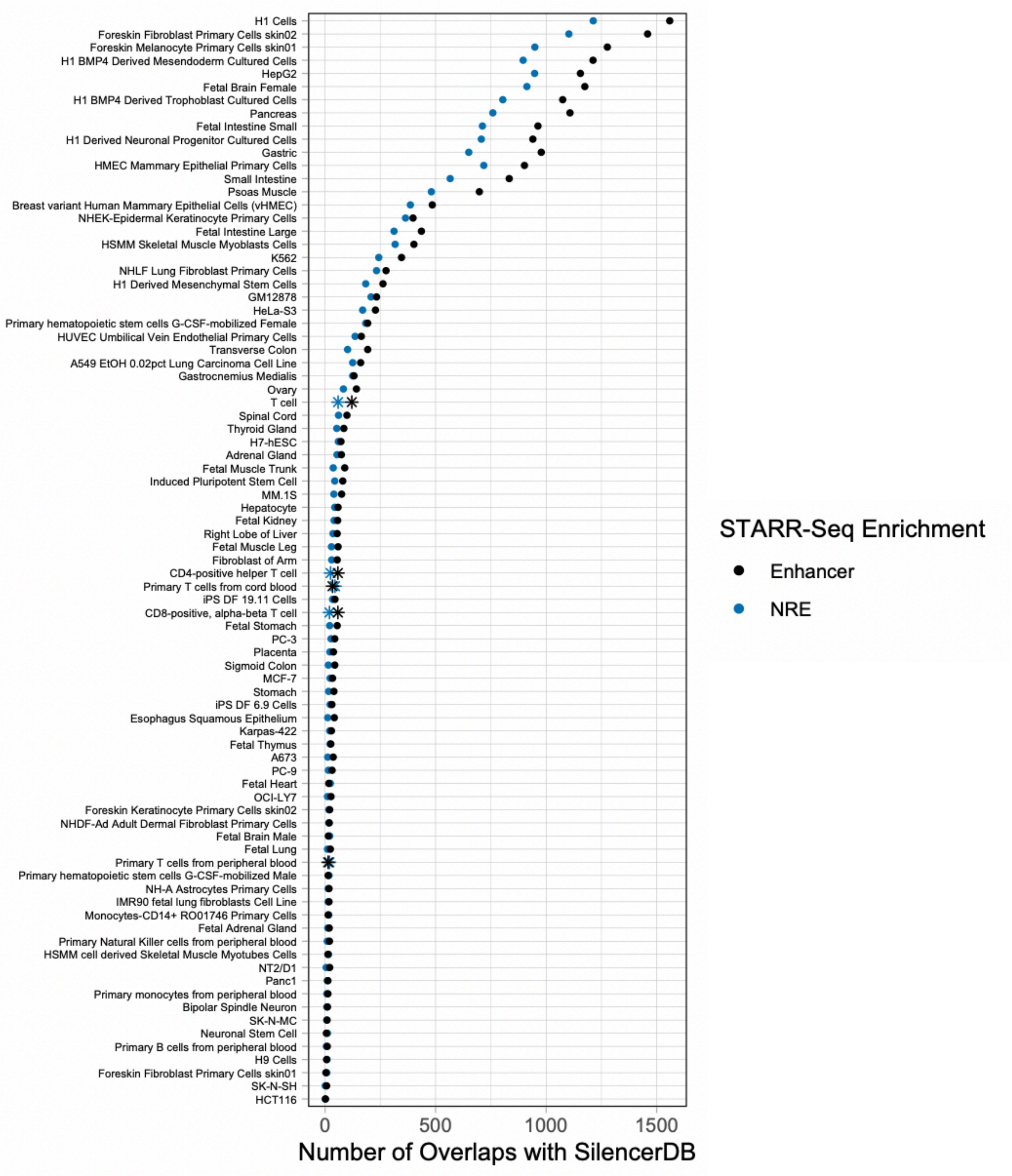

Supplemental Figure 6: Silencer Database (SilencerDB) Intersection of Cis-Regulatory Elements. The number of Lenti-STARR-Seq–identified Cis-regulatory elements (CREs) intersected with predicted silencers in SilencerDB.(7)

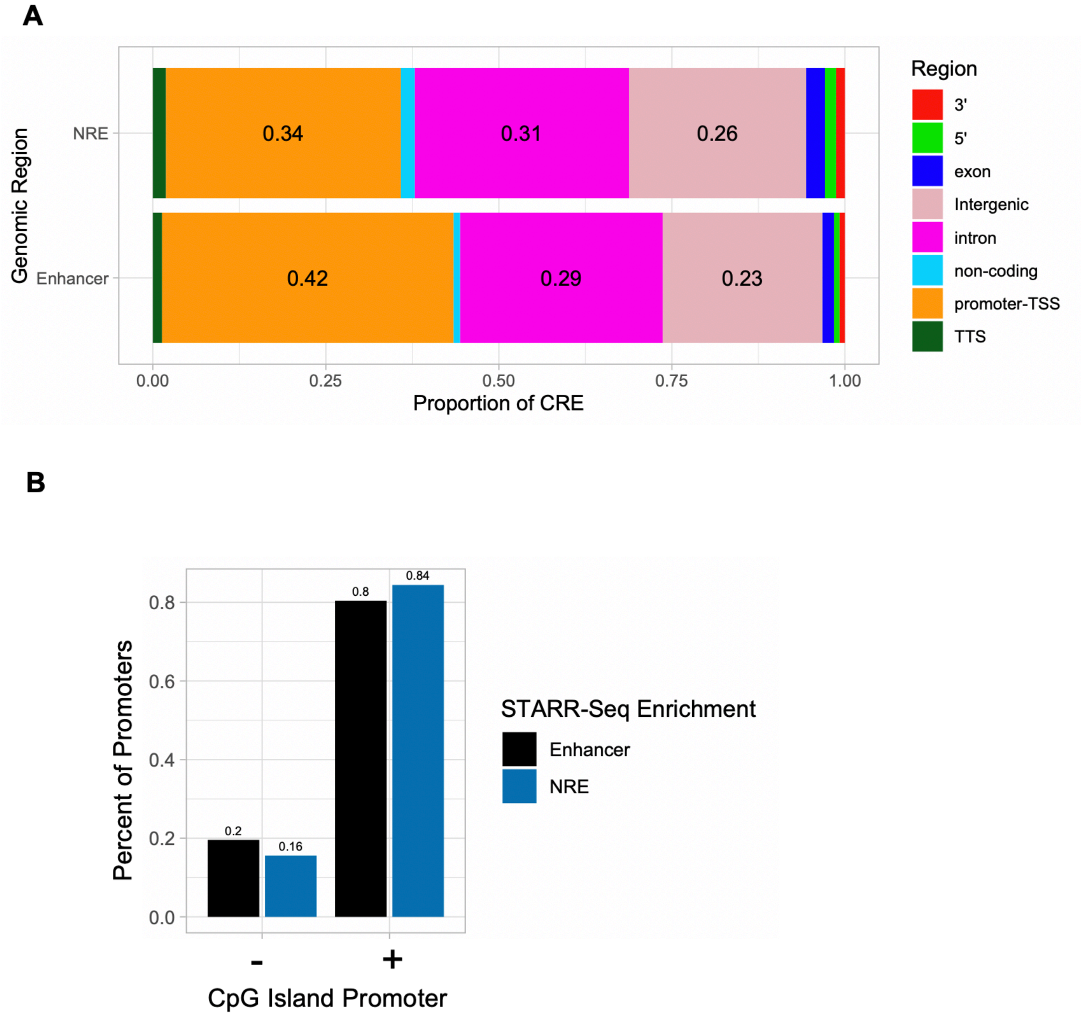

Supplemental Figure 7: CRE Location and CpG Promoter Enrichment. A) The proportion of CRE intersecting each genomic region, annotated by HOMER.(8) **B)** The proportion of CRE intersecting CpG islands [UCSC Genome Browser] within annotated promoters.

Supplementary Figure References:

1. Luo Y, Hitz BC, Gabdank I, Hilton JA, Kagda MS, Lam B, et al. New developments on the Encyclopedia of DNA Elements (ENCODE) data portal. Nucleic Acids Res. 2020 Jan;48(D1):D882–9.
2. Gisselbrecht SS, Palagi A, Kurland J V, Rogers JM, Ozadam H, Zhan Y, et al. Transcriptional Silencers in Drosophila Serve a Dual Role as Transcriptional Enhancers in Alternate Cellular Contexts. Mol Cell [Internet]. 2020 Jan 16;77(2):324-337.e8. Available from: https://doi.org/10.1016/j.molcel.2019.10.004
3. Pang B, Snyder MP. Systematic identification of silencers in human cells. Nat Genet [Internet]. 2020;52(3):254–63. Available from: https://doi.org/10.1038/s41588-020-0578-5
4. Harley JB, Chen X, Pujato M, Miller D, Maddox A, Forney C, et al. Transcription factors operate across disease loci, with EBNA2 implicated in autoimmunity. Nat Genet [Internet]. 2018;50(5):699–707. Available from: https://doi.org/10.1038/s41588-018-0102-3
5. Whyte WA, Orlando DA, Hnisz D, Abraham BJ, Lin CY, Kagey MH, et al. Master transcription factors and mediator establish super-enhancers at key cell identity genes. Cell. 2013 Apr;153(2):307–19.
6. Schep AN, Buenrostro JD, Denny SK, Schwartz K, Sherlock G, Greenleaf WJ. Structured nucleosome fingerprints enable high-resolution mapping of chromatin architecture within regulatory regions. Genome Res. 2015 Nov;25(11):1757–70.
7. Zeng W, Chen S, Cui X, Chen X, Gao Z, Jiang R. SilencerDB: a comprehensive database of silencers. Nucleic Acids Res [Internet]. 2021 Jan 8;49(D1):D221–8. Available from: https://doi.org/10.1093/nar/gkaa839
8. Heinz S, Benner C, Spann N, Bertolino E, Lin YC, Laslo P, et al. Simple combinations of lineage-determining transcription factors prime cis-regulatory elements required for macrophage and B cell identities. Mol Cell. 2010 May;38(4):576–89.
